# Solar Panels as Novel Nest Sites for the European Paper Wasp *Polistes dominula*

**DOI:** 10.1002/ece3.70608

**Published:** 2024-11-20

**Authors:** Nicholas E. Tew, Michael O. Levin, Rebecca R. Hernandez

**Affiliations:** ^1^ Department of Land, Air and Water Resources University of California Davis California USA; ^2^ Wild Energy Center, University of California Davis California USA; ^3^ Ecology, Evolution and Environmental Biology Department Columbia University New York New York USA

**Keywords:** invasive species, nesting behaviour, paper wasp, photovoltaic, *Polistes*, solar energy

## Abstract

Solar energy facilities are rapidly expanding in their land‐use footprint worldwide, with significant implications for biodiversity. Although the impacts of conventional solar development are often negative for biodiversity, it is possible for some species to take advantage of the novel anthropogenic structures and microhabitats provided by solar facilities. We describe the frequent nesting of non‐native European paper wasps (*Polistes dominula*) at two solar facilities in the Central Valley of California (USA), conducting nest censuses to further investigate population density and nest siting behaviour. Active nests were found to occur at a density of 10–23 per hectare of solar facility, and paper wasps had a preference for nesting in sheltered metal torque tubes compared with the more exposed undersides of photovoltaic panels. Our study shows that *P. dominula* might benefit from the construction of solar energy facilities, which could have a variety of impacts on native species and surrounding agriculture that warrant further study. The European paper wasp therefore provides an example to illustrate the potential for a varied and relatively unpredictable set of ecological outcomes to follow land‐use change resulting from solar energy development.

## Graphical abstract

Our study describes and quantifies the frequent nesting of European paper wasps on solar panels and supporting infrastructure in the Central Valley of California. This non‐native invertebrate species is likely to benefit from the construction of solar energy facilities, which could have a variety of impacts on native species and surrounding agriculture that warrant further study.

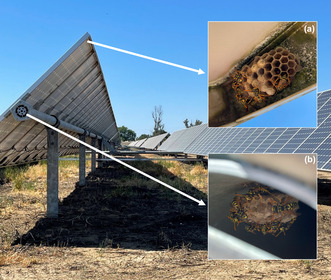

## 1 Introduction

Solar energy is a rapidly growing renewable source of electricity generation worldwide (IEA 2023), but requires large areas of land for photovoltaic (PV) panels (Hernandez et al. 2014). In the USA, large, ground‐mounted photovoltaic (GPV) facilities already cover at least 1300 km^2^ (Fujita et al. 2023), a footprint that could grow to 40,000 km^2^ by 2050 (DOE 2023). The construction of GPV facilities is typically associated with significant changes to land cover (e.g., removal of vegetation and topsoil) and landscape permeability (e.g., construction of fences and roads), with subsequent vegetation management (e.g., herbicide use, mowing or grazing) affecting the nature of plant growth (Hernandez et al. 2014; McCall et al. 2023). Such changes are likely to have significant effects on local biodiversity, altering plant and animal communities, to the benefit of some species (‘winners’) and the detriment of others (‘losers’) (Hernandez et al. 2014; Lafitte et al. 2023). In addition, the physical infrastructure associated with GPV facilities, including PV panels and metal racking systems, may create novel microhabitats used by animals and plants, such as places for birds to perch and nest (Hernandez et al. 2014; Visser et al. 2019).

In two GPV facilities in the Central Valley of California, we noticed the frequent siting of European paper wasp (*Polistes dominula*) nests on PV panels and their supporting infrastructure. These two facilities represent the only solar sites to which we had field access in summer 2024, and nests were found at both. *Polistes dominula* is an introduced social wasp, native to Eurasia and North Africa, which is now widely distributed across North America and considered to be an invasive species (Cervo, Zacchi, and Turillazzi 2000). These wasps have a generalist diet, provisioning larvae with caterpillars and other insects, while adults obtain sugar from sources such as floral nectar, fruit and honeydew (Liebert et al. 2006). Impacts on local ecosystems may include the displacement of North American native paper wasps (especially *Polistes fuscatus* ) and the predation of significant numbers of insects, although the latter may also represent a benefit for the control of agricultural and horticultural pests in certain situations (Baker and Potter 2020; Liebert et al. 2006). *Polistes dominula* naturally builds paper nests on vegetation, but this species also shows an extremely high affinity for nesting on anthropogenic structures, including buildings, fences and machinery (Höcherl and Tautz 2015; Pérez‐Bote and Mora‐Rubio 2020). Given our observations of wasps nesting at solar facilities, we decided to investigate their population density and nest siting behaviour to gain a deeper understanding of their propensity to thrive in this expanding anthropogenic land use. To our knowledge, this is the first published study describing the use of solar panels as nest sites for paper wasps.

## 2 Materials and Methods

**FIGURE 1:**
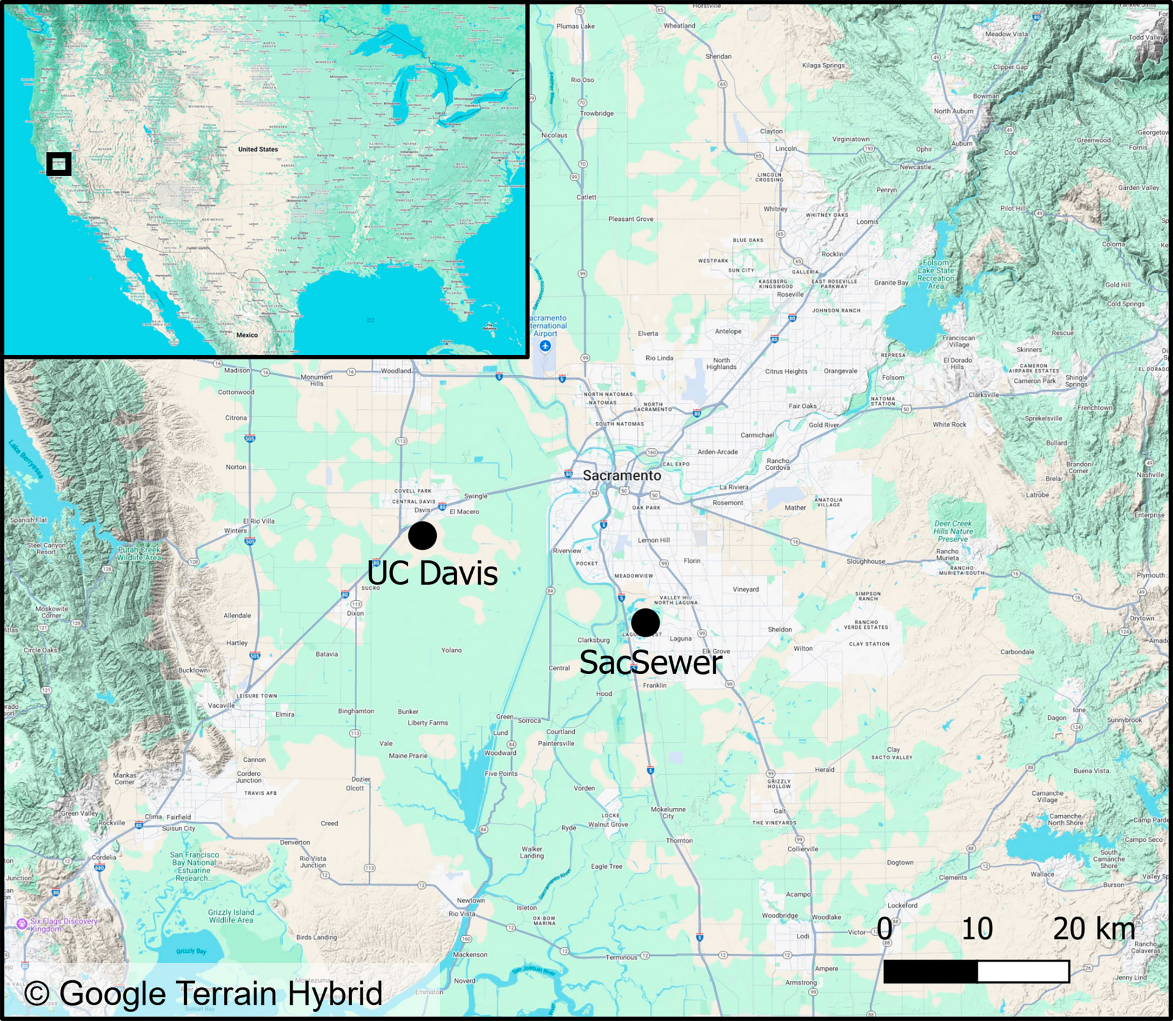
The locations of the two study sites in which paper wasp nests were counted, within the Central Valley of California, USA.

To estimate nest density at the two GPV facilities, we conducted censuses by counting all nests of *P. dominula* along a randomly selected set of PV panel strings (rows of panels) in summer 2024. The University of California's 16 megawatt (MW) GPV facility in Davis (UC Davis facility) is sited on 23 ha of land and includes 936 strings, each containing 40 single‐axis tracking PV panels (Figure 1). For each census, 47 panel strings (5% of the total) were counted along their entire length between 09:30 and 14:30 on June 7th, July 9th and August 1st. The UC Davis facility is surrounded on three sides by agricultural crop fields, with a tree‐lined creek to the south. The Sacramento Area Sewer District EchoWater Resource Recovery Facility's 4‐MW GPV facility in Elk Grove (SacSewer facility) spans eight hectares of land and includes 342 strings, each containing 38 single‐axis tracking PV panels (Figure 1). For each census, 35 panel strings (10% of the total) were counted along their entire length between 08:40 and 11:30 on July 16th and August 21st. The SacSewer facility is surrounded by livestock pasture, with small, wooded areas and residential neighbourhoods nearby.

Nests were categorised as active if at least one live adult wasp was present and inactive if no live adult wasps were present at the time of counting. PV panel strings were visually inspected for nests, with their location on the structure also recorded. Reported values are likely an underestimate given that we could not fully examine hollow elements of the PV string infrastructure (e.g., metal torque tubes within the racking infrastructure that support the panels). We extrapolated nest counts up from our sample of panel strings to estimate facility‐level nest numbers, multiplying by the relevant factor reflecting sampling completeness at each site (936/47 or ∼19.9 for the UC Davis site and 342/35 or ∼9.8 for the SacSewer site). During the July and August censuses, we also recorded the number of live wasps per active nest at both facilities by visually counting wasps on each active nest we encountered, where visibility permitted complete counting.

## 3 Results

**FIGURE 2:**
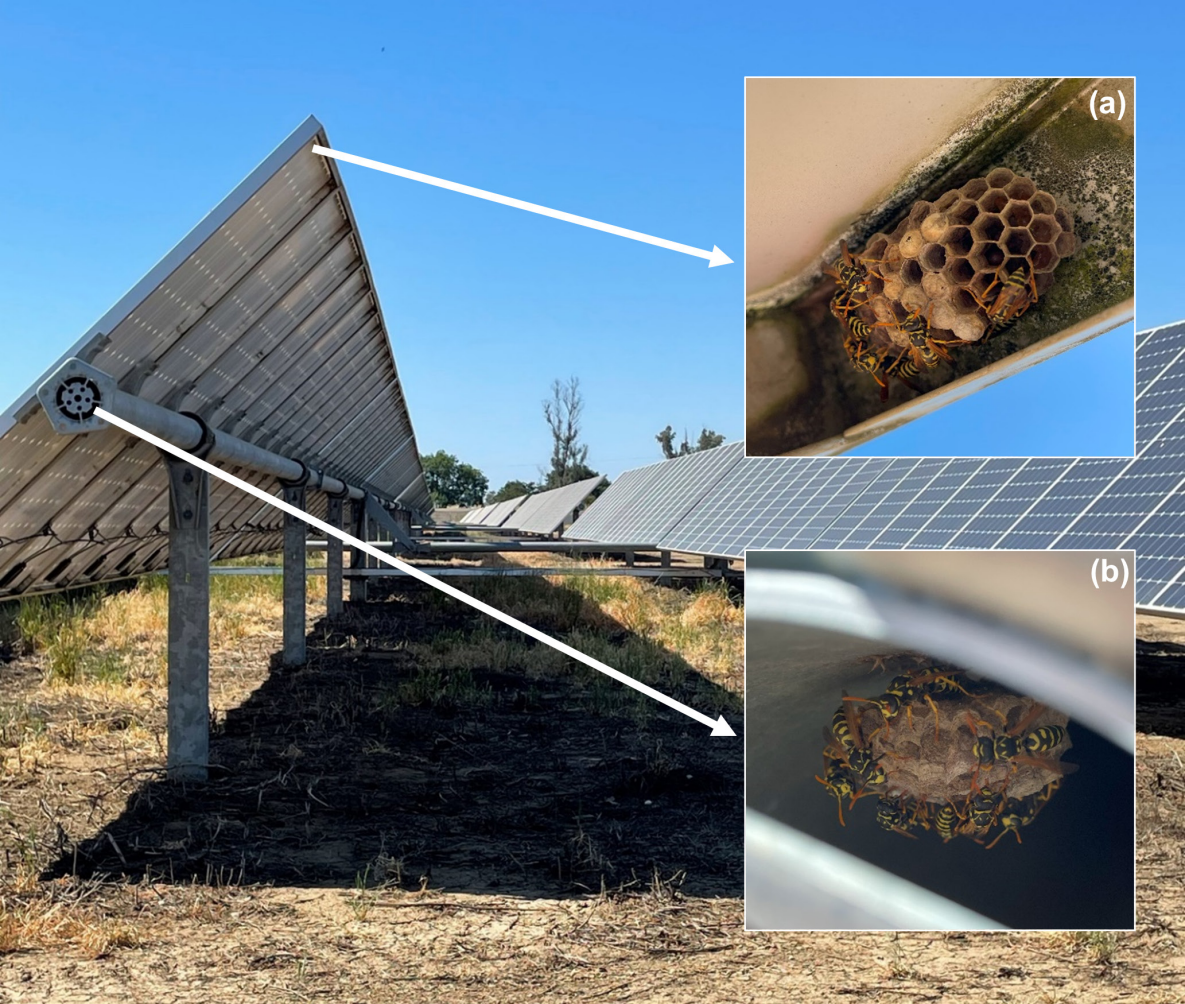
European paper wasps (*Polistes dominula*) nesting on solar infrastructure at the UC Davis solar facility (Davis, California, USA). Nests are constructed in two locations: (a) on the underside of photovoltaic panels and (b) inside metal torque tubes. Photo credit: Nicholas Tew.

We extrapolated our nest counts (Table 1) to estimate 239–518 active nests across the entire UC Davis facility (10–23 per hectare) and 78–127 across the SacSewer facility (10–16 per hectare). We found a mean of 9.7 (± 1.4 SEM) wasps per nest (*n* = 35 counted nests, range 1–36 wasps), providing an estimate of 94–217 adult wasps per hectare of solar facility. Nests were found in two different locations on the solar infrastructure (Figure 2): (1) on the underside of panels (typically in corners and around the edges) and (2) at the end of hollow metal torque tubes running beneath a string of panels. In total, 66% (47/71) of the active nests counted were located within the metal tube, with 34% (24/71) on the underside of panels. For inactive nests, 68% (198/292) were located on the underside of panels, with 32% (94/292) within the metal tube.

**TABLE 1:** Summary statistics of the nest censuses at the UC Davis and SacSewer solar facilities.

Solar facility	Census date	Percentage of panels surveyed	Active nests	Inactive nests	Total active nests (estimated)	Total inactive nests (estimated)
UC Davis	07 June	5	26	109	518	2171
UC Davis	09 July	5	12	75	239	1494
UC Davis	01 August	5	12	75	239	1494
SacSewer	16 July	10	8	13	78	127
SacSewer	21 August	10	13	20	127	195

## 4 Discussion

We show the use of solar infrastructure for nesting by the European paper wasp at two GPV solar energy facilities in the Central Valley of California. In addition, we contacted other researchers in the field, who confirmed sighting European paper wasps nesting on GPV infrastructure in the states of Colorado, Ohio and Wisconsin, as well as the North American native paper wasp *Polistes fuscatus* in Ohio. It is therefore likely that *P. dominula* commonly nests on solar infrastructure where the two co‐occur, and solar facilities may be sites of intra‐generic competition between *Polistes* species, supporting both native and non‐native wasps. Two thirds of all active nests were sited at the ends of the hollow metal torque tube behind a grating or cover, despite this location comprising a small fraction of the total surface area available for nesting (Figure 2). This preference may result from a lower risk of nest predation by birds in such a sheltered location (Cervo, Zacchi, and Turillazzi 2000). Torque tubes often contain small holes throughout their length, so the true number of nests sited within them could be much greater than our estimate, which was based on those that could be readily observed. Differences in solar technology and infrastructure design affect the availability of nesting locations for paper wasps, as well as our ability to observe them. For example, torque tubes are found in tracking PV systems (where they act as a pivot), such as at the UC Davis and SacSewer facilities, but not in fixed systems.

Our observations provide an example of one invertebrate species that could benefit from the increasing development of GPV facilities in the USA, demonstrating that there may be ‘winners’ from solar energy‐driven land‐use change. It is important to note that ‘winners’ might disproportionately be generalist non‐native species capable of taking advantage of the disturbance and novel microhabitats created by GPV development, such as in the case of *P. dominula*. Landscapes dominated by intensive agriculture, where GPV facilities are typically sited (Walston et al. 2021), have relatively little high‐value nesting habitat for paper wasps (e.g., tall, woody vegetation and built structures). As a result, certain types of GPV infrastructure may have the potential to facilitate the spread and increase the local population size of *P. dominula* across the USA. Information about how the local abundance of paper wasps varies with distance from a GPV facility would have helped to clarify the extent to which they are population hotspots for *P. dominula* and the potential population‐limiting effect of nest site availability (e.g., as opposed to food resources). However, sampling wasps away from their nests was challenging as traps (baited with apple, fermented apple juice or chicken) were unsuccessful in capturing wasps and floral visitation rates were too low to yield useful data. Further investigation would be of benefit into how paper wasp nest density compares between solar facilities and other habitats, whether nest site availability directly limits their populations and whether GPV facilities might even act as ecological traps.

If GPV facilities are responsible for increasing the local population size, or at least abundance, of European paper wasps, this could have a variety of effects on the local ecosystem and surrounding agriculture. As a predator of caterpillars and other herbivorous invertebrate species, *P. dominula* could increase nearby crop yields by providing a pest control service (Liebert et al. 2006). Various studies have discussed or modelled the potential beneficial spillover effect of pollination services from GPV facilities to surrounding agriculture (Armstrong et al. 2021; Dolezal, Torres, and O'Neal 2021; Mishra et al. 2023; Walston et al. 2018), but equivalent research is lacking for pest control. A few 100 active *P. dominula* nests per GPV facility may harbour in total just a few 1000 wasps at their peak size, which is roughly equivalent to a single yellowjacket (*Vespula* species) nest (Kasper, Reeson, and Austin 2008). The strength of predation provided by paper wasps nesting at GPV facilities may therefore be relatively modest, but the principle that solar facilities could provide a pest control service that spills over into surrounding farmland warrants further study. In particular, facilities where vegetation management promotes native, diverse forbs and grasses beneath and between panel strings are likely to host significant populations of native predatory insects as well as pollinators (Blaydes et al. 2024; Walston et al. 2024). Paper wasps themselves regularly visit flowers for nectar, thus could also aid in the pollination of nearby flowering crops, with *P. dominula* shown to be an effective pollinator of milkweed in North America, comparable to the native *P. fuscatus* (Rafferty and Ives 2012). In addition, paper wasps might be of benefit to local bird populations, acting as a new food source (Cervo, Zacchi, and Turillazzi 2000). On the other hand, increased populations of this non‐native wasp could reduce native insect numbers, predate declining species such as caterpillars of the monarch butterfly (Baker and Potter 2020) and even damage agricultural production through the consumption or spoiling of fruit (Madden et al. 2017). *Polistes dominula* therefore provides just one example to illustrate the potential for a varied and relatively unpredictable set of ecological outcomes to follow land‐use change resulting from solar energy development.

## Author Contributions

**Nicholas E. Tew:** conceptualization (equal), investigation (lead), methodology (lead), methodology (lead), writing – original draft (lead), writing – original draft (lead), writing – review and editing (equal), writing – review and editing (equal). **Michael O. Levin:** funding acquisition (supporting), writing – review and editing (equal). **Rebecca R. Hernandez:** conceptualization (equal), funding acquisition (lead), project administration (lead), writing – original draft (supporting), writing – review and editing (equal).

## Conflicts of Interest

The authors declare no conflicts of interest.

## Data Availability

Data available from the Dryad Digital Repository https://doi.org/10.5061/dryad.0vt4b8h80 (Tew et al. 2024).
